# *In vitro* modeling of the interaction between human epithelial cells and lymphocytes upon influenza infection

**DOI:** 10.1111/irv.12394

**Published:** 2016-05-17

**Authors:** Natalia A. Ilyushina, Peter F. Wright

**Affiliations:** ^1^ Center for Drug Evaluation and ResearchFood and Drug AdministrationSilver SpringMDUSA; ^2^ Department of PediatricsGeisel School of Medicine at DartmouthLebanonNHUSA

**Keywords:** Cytotoxic T lymphocytes, influenza virus, lysis, nasal human epithelial cells

## Abstract

Influenza viruses are a continuous threat to humans because of their ability to cross species barriers and adapt to new hosts. Data from murine studies, along with limited human data, suggest that CD8^+^ cytotoxic T lymphocytes (CTL) that recognize conserved epitopes of structural influenza proteins are the main mediators of influenza virus clearance. Additionally, the fact that many CTLs recognize epitopes shared between different influenza strains offers the potential for broad cross‐strain immunity. However, the mechanisms of cellular immunity against influenza viruses are poorly defined in humans, where the CTL response has been hard to measure and interpret. We developed a novel CTL assay that utilizes fully differentiated nasal human epithelial cells taken from volunteers as permissive targets for autologous peripheral blood‐derived influenza virus‐specific cytotoxic T lymphocytes. This *in vitro* system of human lymphocyte–epithelial cell co‐cultures can be considered as the closest approximation to events *in vivo* and can be employed for studying the interactions between the pathogen and human host. Modeling of the natural interaction process between the primary cell type that supports the productive replication of influenza and immune cells may allow us to put in perspective CTLs as a correlate of immunity to influenza in humans.

## Introduction

Influenza viruses are a continuous threat to humans because of their ability to cross species barriers and adapt to new hosts. It has been estimated that 5–15% of the world population are infected during annual outbreaks and that these infections result in one million deaths every year.^1^ Influenza virions infect the cells of respiratory pseudostratified columnar epithelium consisting of a single layer of three major cells types, namely ciliated, goblet, and Clara cells.^2^ Data from murine studies, along with limited human data, suggest that CD8^+^ cytotoxic T lymphocytes (CTL) that recognize conserved epitopes of structural influenza proteins are the main mediators of influenza virus clearance.^3^, ^4^, ^5^, ^6^, ^7^ Additionally, the fact that many CTLs recognize epitopes shared between different influenza strains offers the potential for broad cross‐strain immunity.^8^, ^9^, ^10^ However, the mechanisms of cellular immunity against influenza viruses are poorly defined in humans, where the CTL response has been hard to measure and interpret.^4^, ^8^, ^9^, ^11^ In the present study, we developed a novel CTL assay that utilizes fully differentiated nasal human epithelial (NHE) cells taken from volunteers as permissive targets for autologous peripheral blood‐derived influenza virus‐specific cytotoxic T lymphocytes. This *in vitro* system of human lymphocyte–epithelial cell co‐cultures can be considered as the closest approximation to events *in vivo* and can be employed for studying the interactions between the pathogen and human host.

## Methods

### Cells and viruses

Madin–Darby canine kidney (MDCK) cells were obtained from the American Type Culture Collection and maintained as previously described.^12^ Nasal epithelial (obtained by nasal swabbing) and peripheral blood mononuclear cells (PBMCs) were obtained from healthy adult volunteers. Informed consent was obtained from all volunteers using protocols and consent forms approved by the Geisel School of Medicine investigational review board (Dartmouth College). A total of four participants (three men and one woman, average age ~59 years) were enrolled in the study. All donors were seropositive to A/California/07/09 (H1N1) virus (mean hemagglutination inhibition titer ~1/32).

Primary NHE cells of passage 2 were grown either in tissue culture flasks (*i.e*., non‐differentiated cells) or on membrane supports (6·5‐mm Transwell; Corning Inc., Corning, NY, USA) at the air–liquid interface in hormone‐ and growth factor‐supplemented serum‐free medium and fully differentiated 4‐ to 8‐week‐old cultures (~10^4^ cells/insert) were used for the experiments.^13^ PBMCs were cryopreserved after separation on a Ficoll–Hypaque gradient (Sigma‐Aldrich, St. Louis, MO, USA).

The influenza viruses, A/California/07/09 (H1N1) and A/equine/Tennessee/5/86 (H3N8), were obtained from the World Health Organization's collaborating laboratories.

### Assessment of influenza viral growth

Infectivity and viral yields were determined in MDCK cells by plaque assay and by 50% tissue culture infectious dose (TCID_50_) assay as described previously.^12^ Briefly, confluent MDCK cells were incubated at 37°C for 1 h with 10‐fold serial dilutions of virus. The cells were then washed and overlaid with minimal essential medium containing 0·3% bovine serum albumin and 0·9% Bacto agar and incubated at 37°C for 72 h. Plaques were stained with 0·1% crystal violet solution containing 10% formaldehyde, and virus yield was expressed as log_10_ plaque‐forming units (PFU)/ml. In the TCID_50_ assay, confluent monolayers of cell cultures growing in 96‐well microplates were inoculated with serial virus dilutions in the presence of trypsin. After 3 days, virus was titrated by hemagglutination assay, and virus titers were expressed as log_10_TCID_50_/ml by the endpoint method of Reed and Muench.^12^, ^14^

### Cytotoxicity assay

T lymphocytes (CD4^+^ and CD8^+^) were generated from PBMCs using human T‐cell enrichment RosetteSep kit (STEMCELL Technologies Inc., Vancouver, BC, Canada). Stimulation of lymphocytes was carried out in 6‐well plates using autologous non‐differentiated NHE cells infected with influenza A viruses. One day after initiation of infection, the stimulator cells were fixed and lymphocytes were added for secondary stimulation. Additionally, autologous T lymphocytes pulsed for 2 h with influenza viruses were also used as stimulator populations. Human rIL‐2 was added on day 3 and the total stimulation interval was 6 days, after which the stimulated lymphocytes were washed and used in a colorimetric cytotoxicity assay (LDH; Roche, Palo Alto, CA, USA). In brief, NHE cultures seeded on Transwell membranes were infected by exposure of the apical side with influenza viruses, and the stimulated lymphocytes were added 2 h post‐infection (hpi) as effector cells, at effector‐to‐target (E/T) ratios ranging from 220:1 to 10:1, and incubated for 6 h. Lactate dehydrogenase, which is released from the cytosol of damaged cells, was measured on a Molecular Devices multilabel reader with a 490‐nm filter (Molecular Devices Inc., Sunnyvale, CA, USA).

Cell viability of stimulated and unstimulated T lymphocytes in co‐culture with H1N1‐infected epithelial cells was measured using CellTiter Aqueous One solution (Promega, Madison, WI, USA).

### Statistical analysis

The levels of viral titers, T‐cell proliferation, and specific lysis were compared by unpaired *t*‐test or analysis of variance (anova). A probability value of 0·05 was prospectively chosen to indicate that the findings of these analyses were not the result of chance alone.

## Results

We first assayed whether PBMCs can function as effector cells. We infected differentiated NHE cells with A/California/07/09 (H1N1) virus at a multiplicity of infection (MOI) of 0·01, and autologous PBMCs (E/T ≈ 100:1) were added either to the basal compartment or to the apical side of the epithelial cells. H1N1 virus replicated to the similar extent at 24 hpi in all the wells, including those that were incubated without PBMCs (data not shown). We next stimulated PBMCs for 6 days with the autologous non‐differentiated epithelial cells that had been pre‐fixed 24 h after initiation of infection with the A/California/07/09 strain. No changes in viral titers were observed between NHE cells incubated in the presence or absence of the stimulated PBMCs at 24 hpi (data not shown).

**Figure 1:**
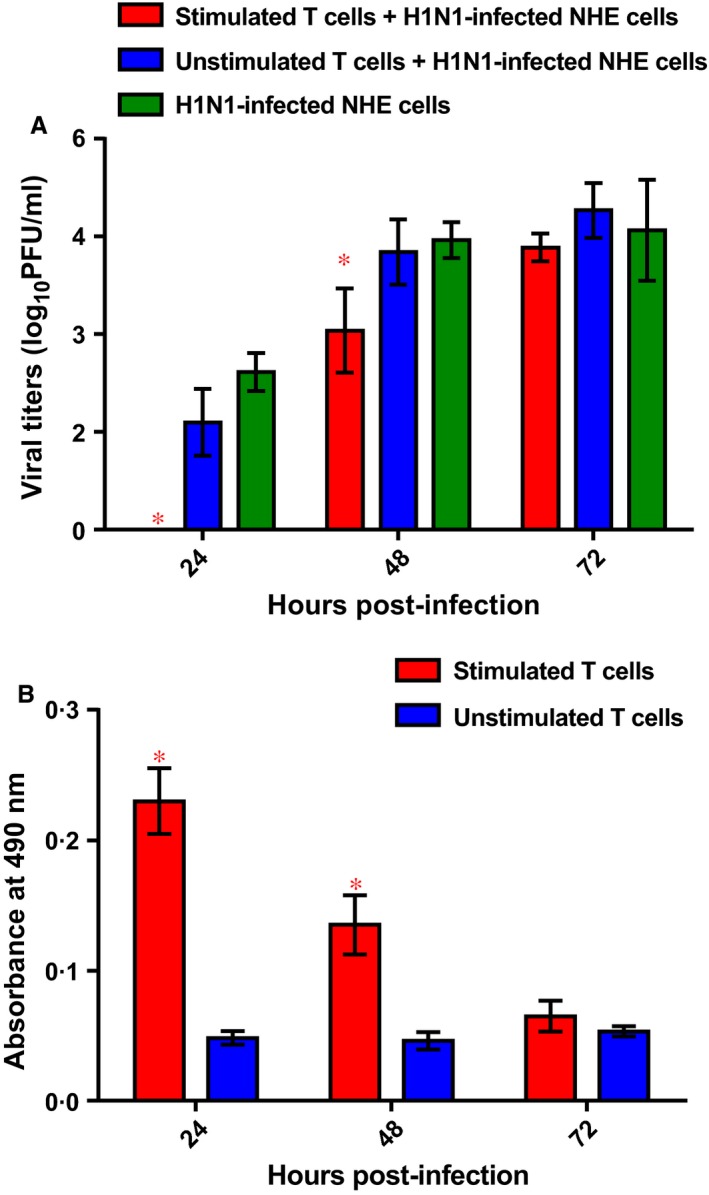
(A) Reduction of viral titers in differentiated nasal epithelial cells infected with A/California/07/09 virus (MOI = 0·01) after addition of autologous stimulated and unstimulated T lymphocytes (E/T ratio ≈ 2·5:1). The T cells were added 2 hpi and were incubated with the nasal human epithelial (NHE) cells for the indicated time intervals. *, *P* < 0·05 compared with control NHEs that were incubated in the absence of effector T cells (one‐way anova). (B) Mean proliferation of stimulated and unstimulated T lymphocytes measured by a 3‐(4,5‐dimethylthiazol‐2‐yl)‐5‐(3‐carboxymethoxyphenyl)‐2‐(4‐sulfophenyl)‐2H‐tetrazolium, inner salt (MTS)‐based assay. The T cells were added 2 hpi and were incubated with the H1N1‐infected NHE cells. The T lymphocytes were collected, and their viability was measured at the indicated time intervals. *, *P* < 0·05 compared with the value for unstimulated T cells by an unpaired *t*‐test.

We next examined whether T lymphocytes isolated from PBMCs could be used as effector cells by comparing viral titers in H1N1‐infected epithelial cells in the presence of stimulated versus unstimulated T cells. Viral titers measured at 24 and 48 hpi were statistically significantly reduced after addition of autologous stimulated T lymphocytes compared with the control NHE cells infected with the A/California/07/09 virus (*P* < 0·05). In contrast, incubation with the unstimulated T cells had no effect on viral replication in the H1N1‐infected NHEs (Figure 1A). We also observed that proliferation of stimulated T lymphocytes was significantly greater than that of unstimulated T cells at 24 and 48 hpi (*P* < 0·05), indicating that the absence of decreased viral growth after 72 h incubation with stimulated T cells was likely due to a loss of T‐cell viability (Figure 1B).

**Figure 2:**
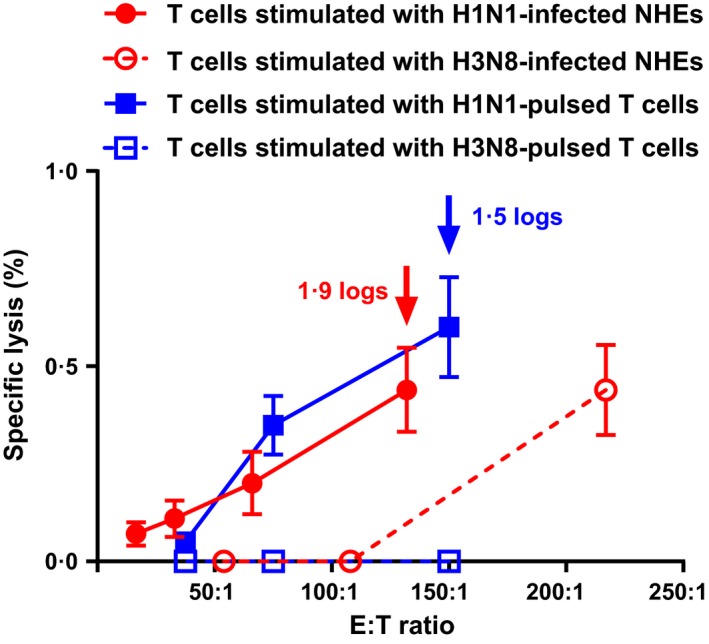
LDH assay based on non‐differentiated nasal epithelial and T cells as stimulators, T lymphocytes as effectors, and infected differentiated nasal human epithelial (NHE) cells as targets. Stimulator cells were incubated with either A/California/07/09 or A/equine/Tennessee/5/86 (H3N8) viruses (MOI = 5), and target cells were infected with A/California/07/09 virus (MOI = 0·01). All cells were isolated from the same adult individual. Cytotoxic T lymphocytes (CTL) response was measured 6 h after the effectors cells were added to the target cells. Arrows and corresponding numbers indicate the statistically significant reduction of viral titers in the target cells at 24 hpi as determined by plaque assay (*P* < 0·05).

**Figure 3:**
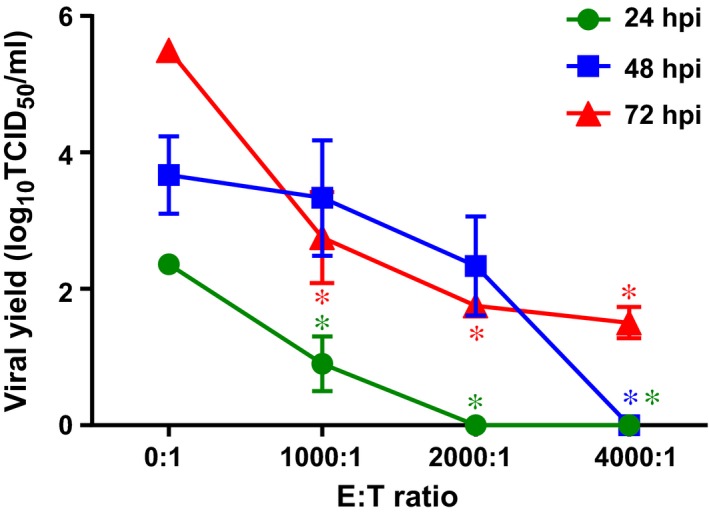
Reduction of viral titers in non‐differentiated nasal epithelial cells infected with A/California/07/09 virus (MOI = 0·01) after addition of autologous stimulated T lymphocytes at indicated E/T ratio. The T cells were added 2 hpi and were incubated with the nasal human epithelial (NHE) cells for the indicated time intervals. *, *P* < 0·05 compared with the control NHEs that were incubated in the absence of the effector T cells (one‐way anova).

We next tested whether the reduction of viral titers by the stimulated T lymphocytes was influenza subtype‐specific in the infected epithelial cells. NHEs were infected with the A/California/07/09 and A/equine/Tennessee/5/86 (H3N8) strains, and the use of these cells as stimulators was compared (Figure 2). Our data showed that despite the fact that the H3N8 virus was able to infect NHE cells,^15^ these cultures were not very effective in stimulating a secondary CTL response (*P* ˃ 0·05). However, some non‐specific lysis that was not associated with the reduced viral titers in the target NHEs was seen when high concentrations of the effector T cells (*i.e*., E/T ≈ 220:1) stimulated with the H3N8‐infected nasal cells were added. We also observed that the H1N1‐pulsed T lymphocytes isolated from the same individual can function as stimulator population, as indicated by the secondary CTL response and reduction of viral titers in the autologous NHEs (Figure2). No specific lysis was detected when heterologous target cells were used (data not shown), suggesting that the CTL response was human leukocyte antigen‐restricted. Additionally, we assayed whether non‐differentiated nasal epithelial cells could be used as the target cells. Our results demonstrated that although the specificity of autologous stimulated T lymphocytes has not been tested, they were able to significantly reduce virus titers at all time points tested (˃1·5 logs, *P* < 0·05; Figure3).

## Discussion

Despite the fact that the CTL response has a well‐characterized role in protection and in recovery from influenza virus infection in the murine model,^3^, ^16^ such a role has not been well documented for humans.^11^ Determination of CTL responses in humans by traditional assays has been restricted by the large volumes of blood needed and the non‐permissivity of the stimulator and target cells used for the assays. Previously, investigations of cell‐mediated immunologic responses to influenza virus have employed either lymphoproliferative responses of peripheral blood lymphocytes, chromium release assay, or IFN‐γ flow cytometric assay/ELISpots.^8^, ^17^, ^18^ However, these assays have not always correlated with serum antibody titers to influenza virus and have limited sensitivity.^8^, ^18^ Furthermore, there is an uncertainty in interpreting the significance of CTL responses measured by these assays because they are often measures of T‐cell activation, but not of effector function. To understand the significance of CTL responses in humans, we must utilize optimal model systems. Here, we developed the novel concept of using differentiated NHE cells as natural and permissive targets for influenza virus cytotoxic T lymphocytes in a CTL assay. This human cell‐culture‐based system of autologous stimulated lymphocyte–epithelial cell co‐cultures provides a valuable tool for the measurement of the levels of CTL immunity as potential protective cell‐mediated immunity at the mucosal level in humans.

Our results suggest that cytotoxic T cells play a role in recovery from influenza infection in humans and that nasal airway epithelia have the capacity to perform as accessory cells *in vitro*. We speculate that NHE cells as the primary cell type infected with influenza A viruses^2^ may function to encounter influenza antigen and directly interact with T cells to initiate local mucosal immune responses. Our data are consistent with the fact that normal human lungs also contain large numbers of resident memory T cells, and these cells are poised to respond to recall antigens previously encountered through lung mucosa.^19^ Overall, we believe that modeling of the natural interaction process between the primary cell type that supports the productive replication of influenza and immune cells can provide critical information about complex networks established between these cells and, therefore, will allow us to put in perspective CTLs as a correlate of immunity to influenza in humans. Understanding the mechanisms by which the inflammatory milieu is created in human conducting airways during influenza disease is likely to lead to the development of immunotherapeutic approaches that will improve both antiviral T‐cell responses and viral clearance.

## Funding

This work was supported by American Recovery and Reinvestment Act funding (contract no. HHSN272200900026C). We are grateful to Dr. Harry Smith for editorial assistance.

## Conflict of interest

The authors do not have any conflict of interests to declare.
